# The complete mitogenome of Smith’s shrew (*Chodsigoa smithii*)

**DOI:** 10.1080/23802359.2019.1640645

**Published:** 2019-07-18

**Authors:** Yingting Pu, Xuemei Tan, Haixue Wei, Changkun Fu, Shunde Chen

**Affiliations:** College of Life Sciences, Sichuan Normal University, Chengdu, China

**Keywords:** Soricomorpha, phylogenetic analysis, evolution

## Abstract

The Smith’s shrew (*Chodsigoa smithii*) belongs to subfamily Soricinae, which is an endemic shrew to China. In this study, we obtained the complete mitochondrial genome of the *C. smithii*. This mitogenome is a circular molecule with 17,108 bp in length, containing 13 protein-coding genes, 22 transfer RNA genes, two ribosome RNA genes, one light strand replication origin (OL), one non-coding region, and with a base composition of 32.5% A, 29.3% T, 24.8% C, and 13.4% G. The nucleotide sequence data of 13 protein-coding genes of *C. smithii* and other 19 Soricomorpha species were used for phylogenetic analyses. Phylogenetic tree shows that Soricinae includes two major phylogenetic lineages. *Chodsigoa smithii* is located as a basal position in tribe Nectogalini.

*Chodsigoa smithii* belongs to subfamily Soricinae, and is an endemic shrew to China, named by Thomas (1911) based on a specimen from Kangding, Sichuan Province, China. It typically occurs in montane broad-leaved forests at elevations of 900–3000 m of Southwest China (Hoffmann and Lunde 2008; Chen et al. 2017). *Chodsigoa smithii* used to include *C. parca* as subspecies (Allen 1923). Subsequently, based on its smaller size and different shape of rostrum (Allen 1923; Hoffmann 1985), Hoffmann (1985) recognized *C. parca* as a distinct species. Due to continuous loss of habitats, this species was classified as Near Threatened on The IUCN Red List (www.iucnredlist.org). Here, we sequenced the whole mitogenome of *C. smithii* (17,108 bp; GenBank accession number: MN038168) and examined its phylogenetic position with other 19 Soricomorpha species.

The individual was captured in Mount Emei, Sichuan Province, China (Latitude: 29°32′38″N, Longitude: 103°20′0″E; H: 2360 m). The specimen was deposited at Sichuan Academy of Forestry (SNU00039). The mitogenome of *Episoriculus macrurus* (NC_029840) was used to design primers for polymerase chain reaction (PCR) and used as template for gene annotation.

The whole mitogenome of *C. smithii* is 17,108 base pairs (bp), including 13 protein-coding genes, 22 transfer RNA genes (tRNA), two ribosome RNA genes(rRNA), one light strand replication origin (OL), and one non-coding region(D-Loop). The entire base composition is as follows: 32.5% A, 29.3% T, 13.4% G, and 24.8% C, with an A + T-rich pattern of the vertebrate mitochondrial genomes. The gene order and gene content of the mitogenome of *C. smithii* is identical to that observed in most other Soricidae (Chen et al. 2015; Kim et al. 2017; Wang et al. 2018).

In order to explore the evolution of subfamily Soricinae, we used 13 protein-coding genes data of mitogenome in *C. smithii* and other 19 Soricomorpha species for the phylogenetic analysis, *Mogera wogura* and *Mogera robusta* were used as outgroups. We used BEASTv1.7 (Drummond et al. 2012) for Bayesian phylogenetic reconstructions, and the best-fit GTR + I + G model of DNA substitution was selected using Akaike Information Criterion (AIC) test in JModelTest 2 (Darriba et al. 2012). The phylogenetic tree is shown in Figure 1, which shows that Soricinae include two distinct phylogenetic lineages (BPP = 1.00). First lineage is containing six species from tribe Nectogalini and Anourosoricini. The second lineage is containing eight species from tribe Blarinellini and Soricini. *C. smithii* is located as a basal position in tribe Nectogalini.

**Figure 1.**
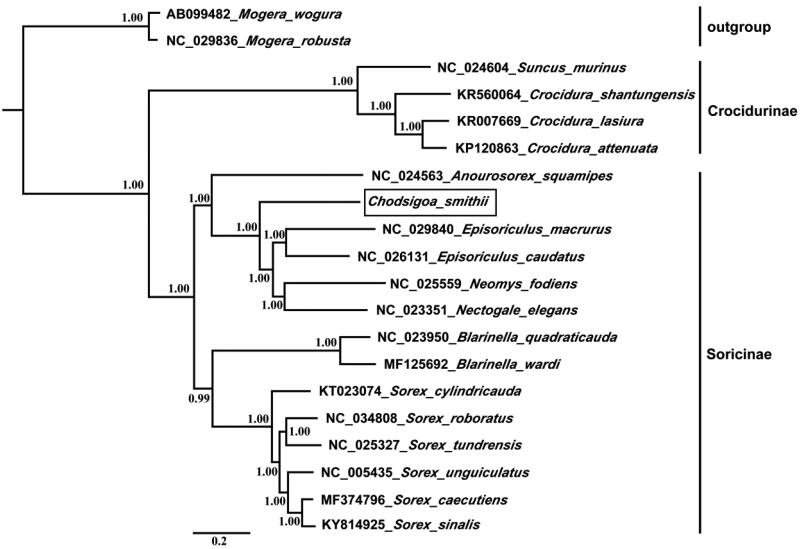
Phylogenetic tree derived from 13 protein-coding gene sequences using BI analysis. Numbers by the nodes indicate Bayesian posterior probabilities.

We expect the study may be useful for phylogenetic and evolution studies of Soricinae, facilitate further investigation of the molecular evolution of genus *Chodsigoa*, and contribute to the conservation of this species.
